# Omicron extensively but incompletely escapes Pfizer BNT162b2 neutralization

**DOI:** 10.1038/s41586-021-04387-1

**Published:** 2021-12-23

**Authors:** Sandile Cele, Laurelle Jackson, David S. Khoury, Khadija Khan, Thandeka Moyo-Gwete, Houriiyah Tegally, James Emmanuel San, Deborah Cromer, Cathrine Scheepers, Daniel G. Amoako, Farina Karim, Mallory Bernstein, Gila Lustig, Derseree Archary, Muneerah Smith, Yashica Ganga, Zesuliwe Jule, Kajal Reedoy, Shi-Hsia Hwa, Jennifer Giandhari, Jonathan M. Blackburn, Bernadett I. Gosnell, Salim S. Abdool Karim, Willem Hanekom, NGS-SA, Mary-Ann Davies, Marvin Hsiao, Darren Martin, Koleka Mlisana, Constantinos Kurt Wibmer, Carolyn Williamson, Denis York, COMMIT-KZN Team, Rohen Harrichandparsad, Kobus Herbst, Prakash Jeena, Thandeka Khoza, Henrik Kløverpris, Alasdair Leslie, Rajhmun Madansein, Nombulelo Magula, Nithendra Manickchund, Mohlopheni Marakalala, Matilda Mazibuko, Mosa Moshabela, Ntombifuthi Mthabela, Kogie Naidoo, Zaza Ndhlovu, Thumbi Ndung’u, Nokuthula Ngcobo, Kennedy Nyamande, Vinod Patel, Theresa Smit, Adrie Steyn, Emily Wong, Anne von Gottberg, Jinal N. Bhiman, Richard J. Lessells, Mahomed-Yunus S. Moosa, Miles P. Davenport, Tulio de Oliveira, Penny L. Moore, Alex Sigal

**Affiliations:** 1 Africa Health Research Institute, Durban, South Africa; 2 School of Laboratory Medicine and Medical Sciences, University of KwaZulu-Natal, Durban, South Africa; 3 Kirby Institute, University of New South Wales, Sydney, New South Wales Australia; 4 National Institute for Communicable Diseases of the National Health Laboratory Service, Johannesburg, South Africa; 5 SA MRC Antibody Immunity Research Unit, School of Pathology, Faculty of Health Sciences, University of the Witwatersrand, Johannesburg, South Africa; 6 KwaZulu-Natal Research Innovation and Sequencing Platform, Durban, South Africa; 7 Centre for Epidemic Response and Innovation, School of Data Science and Computational Thinking, Stellenbosch University, Stellenbosch, South Africa; 8 Centre for the AIDS Programme of Research in South Africa, Durban, South Africa; 9 Department of Medical Microbiology, University of KwaZulu-Natal, Durban, South Africa; 10 Department of Integrative Biomedical Sciences, Faculty of Health Sciences, University of Cape Town, Cape Town, South Africa; 11 Division of Infection and Immunity, University College London, London, UK; 12 Institute of Infectious Disease and Molecular Medicine, University of Cape Town, Cape Town, South Africa; 13 Department of Infectious Diseases, Nelson R. Mandela School of Clinical Medicine, University of KwaZulu-Natal, Durban, South Africa; 14 Department of Epidemiology, Mailman School of Public Health, Columbia University, New York, NY USA; 15 Department of Global Health, University of Washington, Seattle, WA USA; 16 Max Planck Institute for Infection Biology, Berlin, Germany; 17 Center for Infectious Disease Epidemiology and Research, School of Public Health and Family Medicine, University of Cape Town, Cape Town, South Africa; 18 University of Cape Town/Groote Schuur Complex of the National Health Laboratory Service (NHLS), University of Cape Town, Cape Town, South Africa; 19 Division of Computational Biology, Department of Integrative Biomedical Sciences, University of Cape Town, Cape Town, South Africa; 20 Medical Microbiology Department, University of KwaZulu-Natal, Durban, South Africa; 21 National Health Laboratory Services (NHLS), Durban, South Africa; 22 Wellcome Centre for Infectious Diseases Research in Africa, University of Cape Town, Cape Town, South Africa; 23 Molecular Diagnostics Services, Durban, South Africa; 24 Department of Neurosurgery, University of KwaZulu-Natal, Durban, South Africa; 25 South African Population Research Infrastructure Network, Durban, South Africa; 26 Department of Paediatrics and Child Health, University of KwaZulu-Natal, Durban, South Africa; 27 Department of Immunology and Microbiology, University of Copenhagen, Copenhagen, Denmark; 28 Department of Cardiothoracic Surgery, University of KwaZulu-Natal, Durban, South Africa; 29 Department of Medicine, King Edward VIII Hospital and University of KwaZulu Natal, Durban, South Africa; 30 College of Health Sciences, University of KwaZulu-Natal, Durban, South Africa; 31 Ragon Institute of MGH, MIT and Harvard, Boston, MA USA; 32 HIV Pathogenesis Programme, The Doris Duke Medical Research Institute, University of KwaZulu-Natal, Durban, South Africa; 33 Department of Pulmonology and Critical Care, University of KwaZulu-Natal, Durban, South Africa; 34 Department of Neurology, University of KwaZulu-Natal, Durban, South Africa; 35 Division of Infectious Diseases, University of Alabama at Birmingham, Birmingham, AL USA

**Keywords:** SARS-CoV-2, Antimicrobial responses

## Abstract

The emergence of the SARS-CoV-2 variant of concern Omicron (Pango lineage B.1.1.529), first identified in Botswana and South Africa, may compromise vaccine effectiveness and lead to re-infections^1^. Here we investigated Omicron escape from neutralization by antibodies from South African individuals vaccinated with Pfizer BNT162b2. We used blood samples taken soon after vaccination from individuals who were vaccinated and previously infected with SARS-CoV-2 or vaccinated with no evidence of previous infection. We isolated and sequence-confirmed live Omicron virus from an infected person and observed that Omicron requires the angiotensin-converting enzyme 2 (ACE2) receptor to infect cells. We compared plasma neutralization of Omicron relative to an ancestral SARS-CoV-2 strain and found that neutralization of ancestral virus was much higher in infected and vaccinated individuals compared with the vaccinated-only participants. However, both groups showed a 22-fold reduction in vaccine-elicited neutralization by the Omicron variant. Participants who were vaccinated and had previously been infected exhibited residual neutralization of Omicron similar to the level of neutralization of the ancestral virus observed in the vaccination-only group. These data support the notion that reasonable protection against Omicron may be maintained using vaccination approaches.

## Abstract (web summary)

Plasma from individuals vaccinated with BNT162b2 exhibits 22-fold less neutralization capacity against Omicron (B.1.1.529) than against an ancestral SARS-CoV-2 strain but residual neutralization is maintained in those with high levels of neutralization of ancestral virus.

## Main

The emergence of the Omicron variant of SARS-CoV-2 in November 2021, first identified in South Africa and Botswana, was first described in South Africa^2^, followed shortly afterwards by confirmed transmission in Hong Kong^3^. Owing to the large number of mutations in the spike protein and elsewhere on the virus (https://covdb.stanford.edu/page/mutation-viewer/#omicron), there is concern that this variant will exhibit substantial escape from vaccine-elicited immunity^4,5^. Furthermore, several mutations in the spike receptor-binding domain and S2 fusion domain are predicted to increase transmission^5^.

**Extended Data Fig. 1:**
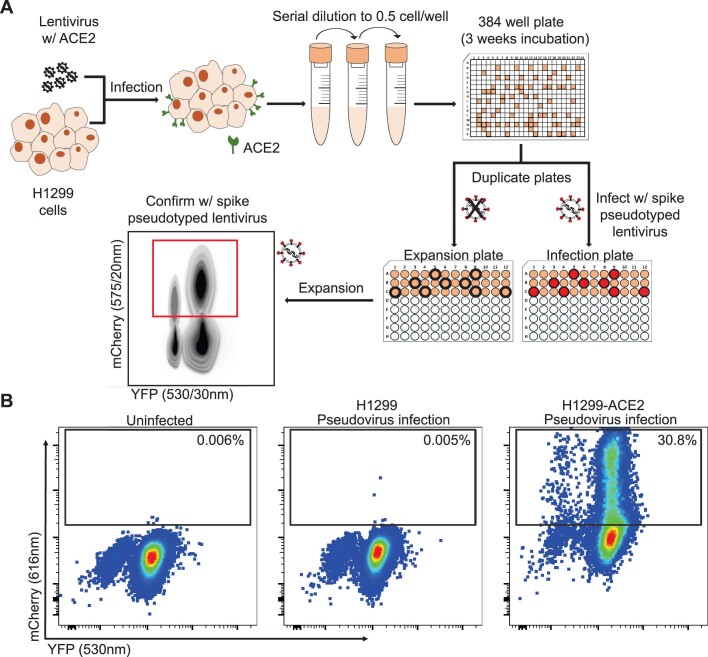
Generation of H1299-ACE2 clonal cell line. (**A**) The H1299 human non-small cell lung carcinoma cell line with YFP labelled histone H2AZ was spinfected with the pHAGE2-EF1a-Int-ACE2 lentivector. Cells were single cell cloned by limiting dilution in a 384-well plate. Clones were expanded into duplicate 96-well plates, where one plate was used to select infectable clones based on mCherry signal from infection with SARS-CoV-2 mCherry expressing spike pseudotyped lentivirus. Clones were chosen based on infectability and expanded from the non-infected replicate 96-well plate. (**B**) Flow cytometry of SARS-CoV-2 mCherry expressing spike pseudotyped lentivirus infection in H1299-ACE2 cells versus H1299 parental cells.

**Extended Data Fig. 2:**
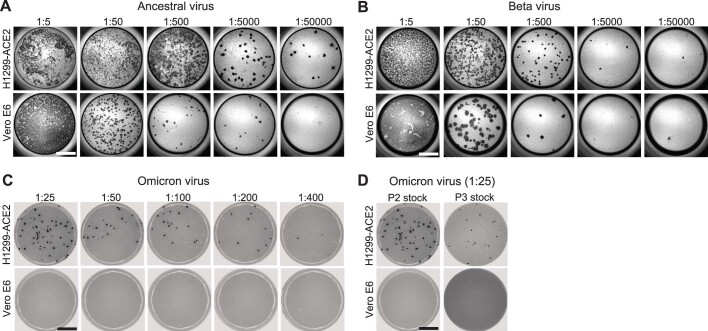
Comparison of SARS-CoV-2 infection in H1299-ACE2 and Vero E6 cells. Both H1299-ACE2 and Vero E6 cells were infected with the same viral stock in the same experiment with D614G virus (**A**) or Beta virus (**B**) and a focus forming assay was performed. (**C**) Focus forming assay with stock of Omicron virus isolate on H1299-ACE2 and Vero E6 cells. (**D**) Comparison of passage 2 (P2) and passage 3 (P3) stock, where P3 stock was generated by infection of 1 mL of cell-free P2 stock in 20 mL of Vero E6 cells seeded at 2x10^5^ cells per mL and incubated over 4 days. Numbers above well images denote viral stock dilution. Scale bar is 2 mm.

Here we have used the human lung cell line H1299-ACE2 (Extended Data Fig. 1), which overexpresses the human ACE2 receptor^6^, to both isolate Omicron and test its neutralization by human plasma. We isolated Omicron virus using one passage on H1299-ACE2 cells and a second passage on H1299-ACE2 cells in co-culture with the Vero E6 African green monkey kidney cell line. Sequencing of the isolated virus confirmed it was the Omicron variant bearing the R346K mutation. We observed no mutations introduced in vitro as majority or minority variants (Extended Data Table 1). H1299-ACE2 cells were similar to Vero E6 cells in that they formed infection foci during infection with ancestral D614G and Beta variant viruses; however, the H1299-ACE2 cells formed more foci than unmodified Vero E6 cells (Extended Data Fig. 2a, b). Infection by cell-free Omicron of unmodified Vero E6 cells was inefficient (Extended Data Fig. 2c) and we could not use cell-free Omicron infection in Vero E6 cells to generate a useable virus stock of this isolate (Extended Data Fig. 2d).

**Fig. 1:**
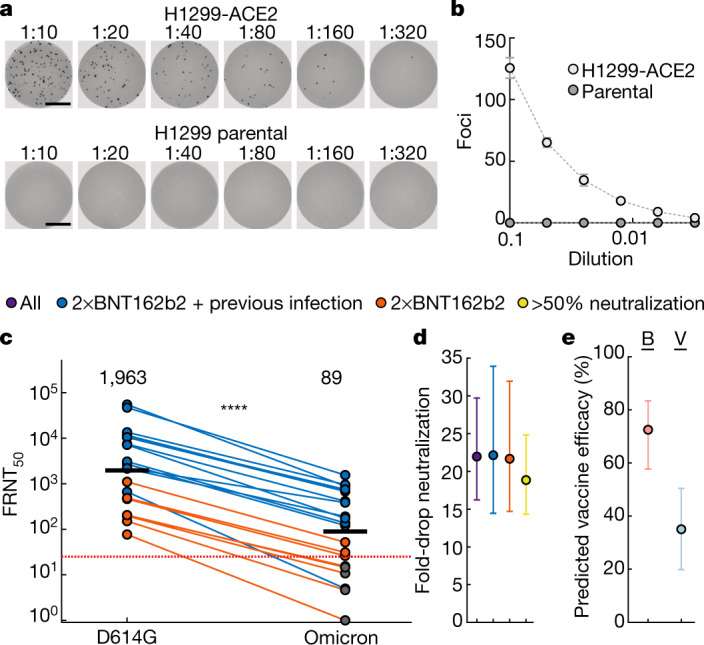
ACE2 dependence and neutralization of the Omicron variant by Pfizer BNT162b2-elicited immunity. **a**, Representative images showing infection foci in wells of a multi-well plate with titration of live SARS-CoV-2 Omicron virus on H1299-ACE2 and H1299 parental cells. Numbers above well images denote viral stock dilution. Scale bars, 2 mm. **b**, Number of foci as a function of Omicron virus stock dilution. Data are mean ± s.d. of six replicates from two independent experiments. **c**, Neutralization of Omicron virus compared with D614G ancestral virus by plasma from participants vaccinated with two doses of BNT162b2 and previously SARS-CoV-2 infected (blue) or uninfected (orange). Numbers in black above each virus strain are geometric mean titres (GMT) of the reciprocal plasma dilution (FRNT_50_) resulting in 50% reduction in infection foci. The red horizontal line denotes the most concentrated plasma used. Twenty-one samples were tested from *n* = 19 participants in 2 independent experiments (*n* = 13 vaccinated and previously infected; *n* = 6 vaccinated only). Grey points denote measurements where 50% neutralization was not achieved with the most concentrated plasma used. *P* = 4.8 × 10^−^^5^, Wilcoxon rank-sum test. **d**, Geometric mean and 95% confidence interval of the fold change in neutralization between ancestral D614G and Omicron neutralization in plasma. Purple denotes all participants, blue denotes vaccinated individuals with previous SARS-CoV-2 infection, orange denotes vaccinated-only individuals, and yellow denotes all participants excluding those in whom 50% neutralization was not achieved. **e**, Mean predicted vaccine efficacy and 95% confidence intervals against symptomatic infection with Omicron using data from previous randomized controlled trials and the 22-fold difference between D614G and Omicron observed in this study^17,18^. Predictions are for vaccinated and boosted (B, red) or vaccinated-only (V, blue) individuals.

We observed that Omicron infected the H1299-ACE2 cells in a concentration-dependent manner but did not infect the parental H1299 cells, indicating that human ACE2 is required for Omicron entry (Fig. 1a, b). We then tested the ability of plasma from individuals vaccinated with BNT162b2 to neutralize Omicron versus ancestral D614G virus in a live virus neutralization assay. We tested plasma samples taken from 19 individuals after they had received 2 doses of BNT162b2 (Extended Data Tables 2, 3), 6 of whom had no previous record of SARS-CoV-2 infection or detectable SARS-CoV-2 nucleocapsid antibodies indicative of previous infection (Methods). We also tested samples from a later time point for two of the vaccinated-only participants (Extended Data Table 3). The previously infected and vaccinated participants were infected with either ancestral SARS-CoV-2 strains or the Delta variant (Extended Data Table 3). To quantify neutralization in the live virus neutralization assay, we calculated the focus reduction neutralization test value (FRNT_50_, the inverse of the plasma dilution required for a 50% reduction in infection focus number).

Consistent with previous studies^7–9^, we observed that individuals who were vaccinated and had previously been infected exhibited higher neutralization capacity for ancestral virus relative to those who were vaccinated only (Fig. 1c). For all participants, the ability to neutralize Omicron was lower than for ancestral virus (Fig. 1c). The geometric mean titre (GMT) FRNT_50_ for all participants was 1,963 for D614G and 89 for Omicron, a 22-fold difference (95% confidence interval 16–30) (Fig. 1d); the fold drop was the same for individuals who were vaccinated and had previously been infected (95% confidence interval 16–34) and in the vaccinated-only group (95% confidence interval 15–32) (Fig. 1d). Six of the samples showed fitted values for 50% Omicron neutralization that corresponded to a plasma concentration higher than the most concentrated plasma tested (a 1:25 dilution). This included the two samples collected at a later time point after vaccination, one of which showed a complete knockout of neutralization activity with Omicron (Fig. 1c, Extended Data Table 3). Excluding these 6 values from the analysis reduced the difference in GMT FRNT_50_ between D614G and Omicron to 19-fold (95% confidence interval 14–25), well within the 95% confidence intervals of the fold differences for the raw values (Fig. 1d). Of note, Omicron virus neutralization by samples from individuals who were previously infected and vaccinated was similar to D614G neutralization by samples from participants vaccinated with two doses of BNT162b2 but not previously infected (Fig. 1c). GMT FRNT_50_ for Omicron in the previously infected and vaccinated group was 305 (95% confidence interval 134–695), whereas GMT FRNT_50_ for ancestral virus in the vaccinated-only group was 263 (95% confidence interval 147–472).

**Extended Data Fig. 3:**
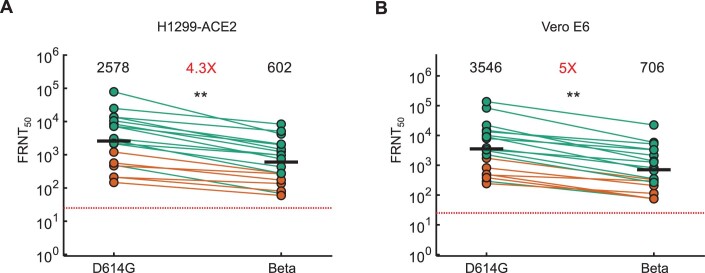
Neutralization of the Beta variant by Pfizer BNT162b2 elicited immunity. Neutralization of the Beta variant virus compared to D614G ancestral virus in H1299-ACE2 (**A**) or Vero E6 cells (**B**) in participants vaccinated with BNT162b2 and infected by SARS-CoV-2 (green) or vaccinated only (orange). Numbers in black above each virus strain are geometric mean titers (GMT) of the reciprocal plasma dilution (FRNT_50_) resulting in 50% reduction in the number of infection foci. Numbers in red denote fold-change in GMT between virus strain on the left and the virus strain on the right of each panel. Red horizontal line denotes most concentrated plasma used. Samples were tested from the n = 19 participants described in Table S2 and S3, where n = 6 were vaccinated only and n = 13 were vaccinated and previously infected. p = 0.006 for both (A) and (B) as determined by the Wilcoxon rank sum test.

We compared these results with neutralization of the Beta variant^6,10–16^ using Beta and D614G virus infection of H1299-ACE2 (Extended Data Fig. 3a) and Vero E6 (Extended Data Fig. 3b) cells. The fold difference relative to the ancestral D614G virus was 4.3 for H1299-ACE2 cells and 5.0 for Vero E6 cells. Thus, results from these two cell lines indicated that Omicron exhibited approximately fourfold greater escape relative to Beta in our assays.

This study was not designed to reliably evaluate vaccine efficacy or protection from severe disease. However, a prediction of vaccine efficacy after a 22-fold drop in neutralization can be made in individuals vaccinated with BNT162b2 and individuals who were both vaccinated and boosted on the basis of data from randomized control trials using a model relating neutralization level to vaccine efficacy^17,18^. Using this model and the differences in neutralization between Omicron and other SARS-CoV-2 strains (Methods), we predict a vaccine efficacy for preventing symptomatic infection by Omicron of 73% (95% confidence interval 58–83%) for vaccinated and boosted individuals, and 35% (95% confidence interval 20–50%) for vaccinated-only individuals; this suggests that Omicron compromises the ability of the vaccine to protect against infection in individuals in the vaccinated-only group but not in vaccinated and boosted individuals (Fig. 1e). We note that these predictions are similar to reports of actual vaccine efficacy in the UK^19^.

Shortly after we released these findings, several other groups have reported similar results^3,20–23^ including Pfizer–BioNTech (https://www.businesswire.com/news/home/20211208005542/en/). These results mirror ours, with large reductions in neutralization of Omicron compared with ancestral virus by vaccine-elicited immunity, neutralizing monoclonal antibodies and plasma from convalescent individuals previously infected with other variants. Notably, the Pfizer–BioNTech study reports that boosting seems to increase neutralization breadth, which reduces the escape by Omicron relative to ancestral virus; these results have been validated independently^21^. Unlike what was reported for boosting, we did not observe a lower fold drop in previously vaccinated and infected  relative to the vaccinated-only participants in this study.

Limitations of this study include the presence of an R346K substitution in our virus stock. This putative escape mutation^24^, which may confer moderate antibody resistance (https://jbloomlab.github.io/SARS2_RBD_Ab_escape_maps/escape-calc/), is not found in the majority of Omicron genomes. In addition, the timing of sample collection soon after vaccination (Supplementary Tables 2, 3) does not account for the waning of neutralization capacity^25,26^.

So far, a milder course of Omicron infection has been observed in South Africa relative to previous infection waves in terms of reported numbers of patients in intensive care units and needing ventilation^27^. Although there may be other unidentified contributing factors that lower pathogenicity^28^, pre-existing immunity would be expected to be higher in the Omicron wave because of vaccination as well as immunity elicited by previous infection during one of three preceding infection waves in South Africa^28^. Therefore, the incomplete Omicron escape from previous immunity described here may be an important factor accounting for the milder course of infection. Despite the extensive neutralization escape of Omicron, residual neutralization levels may still be sufficient to protect from severe disease^17,18^. Other facets of the adaptive immune response elicited by vaccination and previous infection may increase protection. Furthermore, we observed that vaccination combined with previous infection elicits similar neutralization capacity against Omicron as vaccination without previous infection elicits against ancestral virus. This indicates that protection from symptomatic Omicron infection may occur when vaccination is combined with previous infection or boosting. This may explain why Pfizer BNT162b2 vaccination has been shown to substantially decrease the risk of hospital admission caused by Omicron infection in South Africa^29^ and supports the use of further vaccination and boosting to combat Omicron.

## Methods

### Whole-genome sequencing, genome assembly and phylogenetic analysis

cDNA synthesis was performed on the extracted RNA using random primers followed by gene-specific multiplex PCR using the ARTIC V.3 protocol (https://www.protocols.io/view/covid-19-artic-v3-illumina-library-construction-an-bibtkann). In brief, extracted RNAwas converted to cDNA using the Superscript IV First Strand synthesis system (Life Technologies) and random hexamer primers. SARS-CoV-2 whole-genome amplification was performed by multiplex PCR using primers designed using Primal Scheme (http://primal.zibraproject.org/) to generate 400-bp amplicons with an overlap of 70 bp that covers the 30-kb SARS-CoV-2 genome. PCR products were cleaned up using AmpureXP purification beads (Beckman Coulter) and quantified using the Qubit dsDNA High Sensitivity assay on the Qubit 4.0 instrument (Life Technologies). We then used the Illumina Nextera Flex DNA Library Prep kit according to the manufacturer’s protocol to prepare indexed paired-end libraries of genomic DNA. Sequencing libraries were normalized to 4 nM, pooled and denatured with 0.2 N sodium acetate. Then, a 12-pM sample library was spiked with 1% PhiX (a PhiX Control v.3 adaptor-ligated library was used as a control). We sequenced libraries on a 500-cycle v.2 MiSeq Reagent Kit on the Illumina MiSeq instrument (Illumina). We assembled paired-end fastq reads using Genome Detective 1.126 (https://www.genomedetective.com) and the Coronavirus Typing Tool. We polished the initial assembly obtained from Genome Detective by aligning mapped reads to the reference sequences and filtering out low-quality mutations using the bcftools 1.7-2 mpileup method. Mutations were confirmed visually with BAM files using Geneious software (Biomatters). P2 stock was sequenced and confirmed Omicron with the following substitutions: E:T9I, M:D3G, M:Q19E, M:A63T, N:P13L, N:R203K, N:G204R, ORF1a:K856R, ORF1a:L2084I, ORF1a:A2710T, ORF1a:T3255I, ORF1a:P3395H, ORF1a:I3758V, ORF1b:P314L, ORF1b:I1566V, ORF9b:P10S, S:A67V, S:T95I, S:Y145D, S:L212I, S:G339D, S:R346K, S:S371L, S:S373P, S:S375F, S:K417N, S:N440K, S:G446S, S:S477N, S:T478K, S:E484A, S:Q493R, S:G496S, S:Q498R, S:N501Y, S:Y505H, S:T547K, S:D614G, S:H655Y, S:N679K, S:P681H, S:N764K, S:D796Y, S:N856K, S:Q954H, S:N969K and S:L981F. Deletions: N:E31, N:R32, N:S33, ORF1a:S2083, ORF1a:L3674, ORF1a:S3675, ORF1a:G3676, ORF9b:E27, ORF9b:N28, ORF9b:A29, S:H69, S:V70, S:G142, S:V143, S:Y144 and S:N211. The sequence was deposited at GISAID under accession EPI_ISL_7358094.

### SARS-CoV-2 nucleocapsid enzyme-linked immunosorbent assay (ELISA)

Nucleocapsid protein (2 μg ml^−1^) (Biotech Africa; catalogue (cat.) no. BA25-P) was used to coat 96-well, high-binding plates and incubated overnight at 4 °C. The plates were incubated in a blocking buffer consisting of 5% skimmed milk powder, 0.05% Tween 20, 1× PBS. Plasma samples were diluted to a 1:100 dilution in a blocking buffer and added to the plates. Horseradish peroxidase (HRP)-conjugated IgG secondary antibody was diluted to 1:3,000 in blocking buffer and added to the plates followed by tetramethylbenzidine (TMB) peroxidase substrate (Thermo Fisher Scientific). Upon stopping the reaction with 1 M H_2_SO_4_, absorbance was measured at a 450-nm wavelength.

### Cells

Vero E6 cells (ATCC CRL-1586, obtained from Cellonex) were propagated in complete growth medium consisting of Dulbecco’s modified Eagle medium (DMEM) with 10% fetal bovine serum (Hyclone) containing 10 mM of HEPES, 1 mM sodium pyruvate, 2 mM l-glutamine and 0.1 mM nonessential amino acids (Sigma-Aldrich). Vero E6 cells were passaged every 3–4 days. H1299 cell lines were propagated in growth medium consisting of complete Roswell Park Memorial Institute (RPMI) 1640 medium with 10% fetal bovine serum containing 10 mM of HEPES, 1 mM sodium pyruvate, 2 mM l-glutamine and 0.1 mM nonessential amino acids. H1299 cells were passaged every second day. The H1299-E3 (H1299-ACE2, clone E3) cell line was derived from H1299 (CRL-5803) as described in previous work^6^ and Supplementary Fig. 1. In brief, vesicular stomatitis virus G glycoprotein (VSVG) pseudotyped lentivirus containing ACE2 was used to spinfect H1299 cells. ACE-2 transduced H1299 cells (containing an endogenously yellow fluorescent protein labelled histone H2AZ gene^30^) were then subcloned at single-cell density in 96-well plates (Eppendorf) in conditioned medium derived from confluent cells. After 3 weeks, wells were detached using a 0.25% trypsin-EDTA solution (Gibco) and plated in 2 replicate plates, where the first plate was used to determine infectivity and the second was stock. The first plate was screened for the fraction of mCherry-positive cells per cell clone upon infection with a SARS-CoV-2 mCherry expressing spike pseudotyped lentiviral vector. Screening was performed using a Metamorph-controlled (Molecular Devices) Nikon TiE motorized microscope (Nikon) with a 20×, 0.75 NA phase objective, 561-nm laser line, and 607-nm emission filter (Semrock). Images were captured using an 888 EMCCD camera (Andor). The clone with the highest fraction of mCherry expression was expanded from the stock plate and denoted H1299-E3. Infectivity was confirmed with mCherry expressing lentivirus by flow cytometry using a BD Fortessa instrument and analysed using BD FACSDiva Software (BD Biosciences). This clone was used in the outgrowth and focus forming assay. Cell lines have not been authenticated. The cell lines have been tested for mycoplasma contamination and are mycoplasma negative.

### Virus expansion

All work with live virus was performed in biosafety level 3 containment using protocols for SARS-CoV-2 approved by the Africa Health Research Institute Biosafety Committee. ACE2-expressing H1299-E3 cells were seeded at 4.5 × 10^5^ cells in a well on a 6-well plate and incubated for 18–20 h. After one DPBS wash, the sub-confluent cell monolayer was inoculated with 500 μl universal transport medium diluted 1:1 with growth medium filtered through a 0.45-μm filter. Cells were incubated for 1 h. Wells were then filled with 3 ml complete growth medium. After 4 days of infection (completion of passage 1 (P1)), cells were trypsinized, centrifuged at 300*g* for 3 min and resuspended in 4 ml growth medium. Then, 2 ml was added to Vero E6 cells that had been seeded at 2 × 10^5^ cells per ml, 5 ml total, 18–20 h earlier in a T25 flask (approximately1:8 donor-to-target cell dilution ratio) for cell-to-cell infection. The co-culture of ACE2-expressing H1299-E3 and Vero E6 cells was incubated for 1 h and 7 ml of complete growth medium was added to the flask and incubated for 4 days. The viral supernatant (passage 2 (P2) stock) wasused for experiments. Further optimization of the viral outgrowth protocol used for subsequent Omicron isolates showed that addition of 4 ml instead of 2 ml of infected H1299-E3 cells to Vero E6 cells that had been seeded at 2 × 10^5^ cells per ml, 20 ml total, 18–20 h earlier in a T75 flask gave P2 stocks with substantially higher titres that could detectably infect Vero E6 cells. The Omicron virus isolate is available from the authors contingent on verification that it will be received and used in a biosafety level 3 facility.

### Live virus neutralization assay

H1299-E3 cells were plated in a 96-well plate (Corning) at 30,000 cells per well 1 day before infection. Plasma was separated from EDTA-anticoagulated blood by centrifugation at 500*g* for 10 min and stored at −80 °C. Aliquots of plasma samples were heat-inactivated at 56 °C for 30 min and clarified by centrifugation at 10,000*g* for 5 min. Virus stocks were used at approximately 50–100 focus-forming units per microwell and added to diluted plasma. Antibody–virus mixtures were incubated for 1 h at 37 °C, 5% CO_2_. Cells were infected with 100 μl of the virus–antibody mixtures for 1 h, then 100 μl of a 1× RPMI 1640 (Sigma-Aldrich, R6504), 1.5% carboxymethylcellulose (Sigma-Aldrich, C4888) overlay was added without removing the inoculum. Cells were fixed 18 h after infection using 4% PFA (Sigma-Aldrich) for 20 min. Foci were stained with a rabbit anti-spike monoclonal antibody (BS-R2B12, GenScript A02058) at 0.5 μg ml^−1^ in a permeabilization buffer containing 0.1% saponin (Sigma-Aldrich), 0.1% BSA (Sigma-Aldrich) and 0.05% Tween-20 (Sigma-Aldrich) in PBS. Plates were incubated with primary antibody overnight at 4 °C, then washed with wash buffer containing 0.05% Tween-20 in PBS. Secondary goat anti-rabbit HRP conjugated antibody (Abcam ab205718) was added at 1 μg ml^−1^ and incubated for 2 h at room temperature with shaking. TrueBlue peroxidase substrate (SeraCare 5510-0030) was then added at 50 μl per well and incubated for 20 min at room temperature. Plates were imaged in an ImmunoSpot Ultra-V S6-02-6140 Analyzer ELISPOT instrument with BioSpot Professional built-in image analysis (C.T.L).

### Statistics and fitting

All statistics and fitting were performed in MATLAB v.2019b. Neutralization data were fit to:

$${\rm{Tx}}=\frac{1}{1+(D{/{\rm{ID}}}_{50})}$$

Here Tx is the number of foci normalized to the number of foci in the absence of plasma on the same plate at dilution *D* and ID_50_ is the plasma dilution giving 50% neutralization. FRNT_50_ = 1/ID_50_. Values of FRNT_50_ < 1 are set to 1 (undiluted), the lowest measurable value. The most concentrated plasma dilution was 1:25 and therefore FRNT_50_ < 25 were extrapolated. We have marked these values in Fig. 1c and calculate the fold-change FRNT_50_ either for the raw values or for values where FRNT_50_ > 25 in Fig. 1d.

### Estimating vaccine efficacy from neutralization titres

Previously, the fold reduction in neutralization was shown to correlate and predict vaccine efficacy against symptomatic infection with ancestral SARS-CoV-2^18^, and more recently with variants of concern^17^ in data from randomized controlled trials. The model was used here to estimate the vaccine efficacy against Omicron based on the fold drop observed in this study applied to the randomized controlled trial data. In brief, vaccine efficacy (VE) was estimated based on the (log_10_) fold drop in neutralization titre to Omicron (*f*), and the (log_10_) mean neutralization titre as a fold of the mean convalescent titre reported for BNT162b2 in phase I/II trials (*μ*) using the equation:

$${\rm{VE}}(\mu ,f)={\int }_{-\infty }^{\infty }N(x,\mu -f,\sigma )\frac{1}{1+{{\rm{e}}}^{-k(x-{x}_{50})}}\,dx.$$

Here, *N* is the probability density function of a normal distribution with mean *μ* – *f* and standard deviation *σ*, and *k* and *x*_50_ are the parameters of the logistic function relating neutralization to protection for the Pfizer BNT162b2 vaccine which were fitted from randomized controlled trial data: *σ* = 0.46, *k* = 3 and ${x}_{50}={\log }_{10}0.2$ for symptomatic infection^18^. Importantly, $\mu ={\log }_{10}2.4$ for trial participants vaccinated with two doses of BNT162b2, and $\mu ={\log }_{10}12$ for vaccinated and boosted trial participants^17,18^.

### Informed consent and ethical statement

Blood samples were obtained after written informed consent from hospitalized adults with PCR-confirmed SARS-CoV-2 infection and/or vaccinated individuals who were enrolled in a prospective cohort study approved by the Biomedical Research Ethics Committee at the University of KwaZulu–Natal (reference BREC/00001275/2020). Use of residual swab sample was approved by the University of the Witwatersrand Human Research Ethics Committee (HREC) (ref. M210752).

### Reporting summary

Further information on research design is available in the Nature Research Reporting Summary linked to this paper.

## Online content

Any methods, additional references, Nature Research reporting summaries, source data, extended data, supplementary information, acknowledgements, peer review information; details of author contributions and competing interests; and statements of data and code availability are available at 10.1038/s41586-021-04387-1.

### Supplementary information

Reporting Summary

## Figures and Tables

**Extended Data Table 1:**
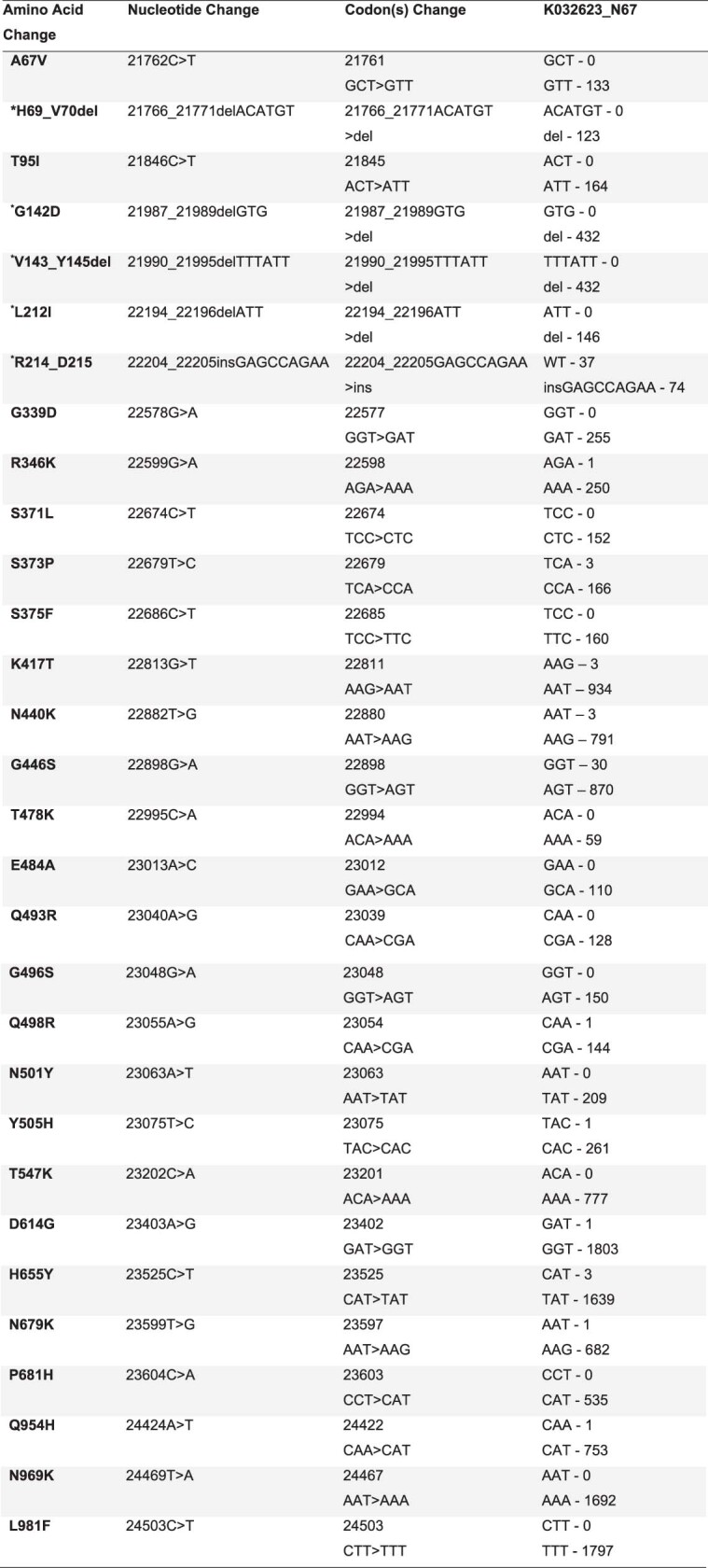
Codon frequency table

This table shows the amino acid change, the nucleotide position of the genome, codon change and the frequency of the codon on the assembled genome.

*Only deletions or insertion where the adjacent codon was preserved were counted; WT - Wild Type, i.e reads without the insertion.

**Extended Data Table 2:**
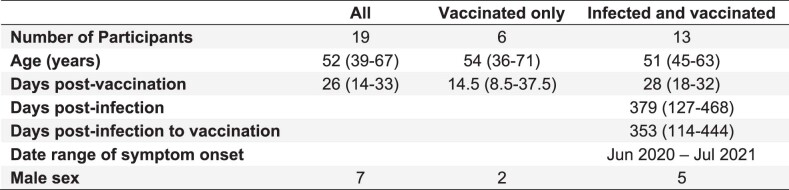
Summary table of participants

All values are median (IQR) and inclusive of all samples used (early and late timepoints for 2 participants).

**Extended Data Table 3:**
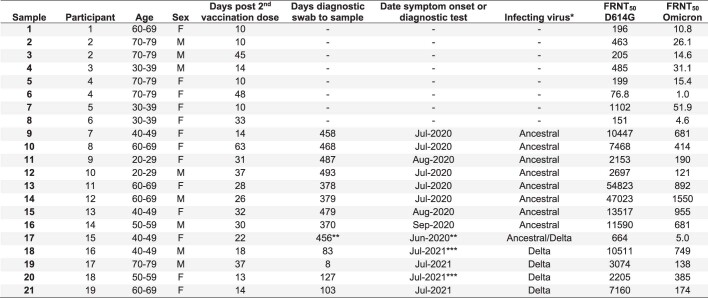
Participant information per sample

*Determined by infection wave in South Africa. First infection wave (April-October 2020) consisted of ancestral strains with the D614G mutation. Third infection wave (April-October 2021) was dominated by the Delta variant. **Participant reinfected during Delta infection wave, sample is taken 3 months post-recovery of Delta infection. Asymptomatic during reinfection. ***Asymptomatic.

## Data Availability

Sequence of outgrown virus has been deposited in GISAID with accession EPI_ISL_7358094. Raw images of the data are available upon reasonable request.

## Code availability

The sequence analysis and visualization pipeline are available on GitHub (https://github.com/nextstrain/ncov). Image analysis and curve fitting scripts in MATLAB v.2019b are available on GitHub (https://github.com/sigallab/NatureMarch2021).
