# Supplementary material for: Omicron extensively but incompletely escapes Pfizer BNT162b2 neutralization
Source: Nature. 2021 Dec 23;602(7898):654–6. doi: 10.1038/s41586-021-04387-1 (PMC8866126; doi:10.1038/s41586-021-04387-1)
Supplement: Supplementary file 1 — Reporting Summary [file 41586_2021_4387_MOESM1_ESM.pdf]

nature portfolio

Corresponding author(s): Alex Sigal
Last updated by author(s): Dec 17, 2021

Reporting Summary

Nature Portfolio wishes to improve the reproducibility of the work that we publish. This form provides structure for consistency and transparency in reporting. For further information on Nature Portfolio policies, see our Editorial Policies and the Editorial Policy Checklist.

Statistics

For all statistical analyses, confirm that the following items are present in the figure legend, table legend, main text, or Methods section.

n/a	Confirmed	
☐	☒	The exact sample size (*n*) for each experimental group/condition, given as a discrete number and unit of measurement
☐	☒	A statement on whether measurements were taken from distinct samples or whether the same sample was measured repeatedly
☐	☒	The statistical test(s) used AND whether they are one- or two-sided<br>*Only common tests should be described solely by name; describe more complex techniques in the Methods section.*
☒	☐	A description of all covariates tested
☒	☐	A description of any assumptions or corrections, such as tests of normality and adjustment for multiple comparisons
☐	☒	A full description of the statistical parameters including central tendency (e.g. means) or other basic estimates (e.g. regression coefficient) AND variation (e.g. standard deviation) or associated estimates of uncertainty (e.g. confidence intervals)
☐	☒	For null hypothesis testing, the test statistic (e.g. *F*, *t*, *r*) with confidence intervals, effect sizes, degrees of freedom and *P* value noted<br>*Give P values as exact values whenever suitable.*
☒	☐	For Bayesian analysis, information on the choice of priors and Markov chain Monte Carlo settings
☒	☐	For hierarchical and complex designs, identification of the appropriate level for tests and full reporting of outcomes
☒	☐	Estimates of effect sizes (e.g. Cohen's *d*, Pearson's *r*), indicating how they were calculated

Our web collection on statistics for biologists contains articles on many of the points above.

Software and code

Policy information about availability of computer code

Data collection	No code used
Data analysis	All statistics and fitting were performed using MATLAB v.2019b.

For manuscripts utilizing custom algorithms or software that are central to the research but not yet described in published literature, software must be made available to editors and reviewers. We strongly encourage code deposition in a community repository (e.g. GitHub). See the Nature Portfolio guidelines for submitting code & software for further information.

Data

Policy information about availability of data

All manuscripts must include a data availability statement. This statement should provide the following information, where applicable:

- Accession codes, unique identifiers, or web links for publicly available datasets
- A description of any restrictions on data availability
- For clinical datasets or third party data, please ensure that the statement adheres to our policy

Sequence was deposited in GISAID, accession: EPI_ISL_7358094. All data are contained in the manuscript.

Field-specific reporting

Please select the one below that is the best fit for your research. If you are not sure, read the appropriate sections before making your selection.

☒ Life sciences ☐ Behavioural & social sciences ☐ Ecological, evolutionary & environmental sciences

For a reference copy of the document with all sections, see nature.com/documents/nr-reporting-summary-flat.pdf

Life sciences study design

All studies must disclose on these points even when the disclosure is negative.

Sample size	Sample size was not pre-determined. We used all the samples we had available which met the inclusion/exclusion criteria.
Data exclusions	We excluded samples from PfizerBNT162b2 vaccinated participants who were previously infected with the Beta variant since we wanted to compare to the Omicron to Beta virus neutralization. We excluded samples positive for SARS-CoV-2 nucleocapsid (ie previously infected) where we could not determine the infecting variant/strain by a time of infection.
Replication	Repeated in an independent experiment on a different day. Geometric mean of replicate samples was used.
Randomization	Groups were determined based on whether
Blinding	No blinding.

Reporting for specific materials, systems and methods

We require information from authors about some types of materials, experimental systems and methods used in many studies. Here, indicate whether each material, system or method listed is relevant to your study. If you are not sure if a list item applies to your research, read the appropriate section before selecting a response.

Materials & experimental systems

n/a	Involved in the study	
☐	☒	Antibodies
☐	☒	Eukaryotic cell lines
☒	☐	Palaeontology and archaeology
☒	☐	Animals and other organisms
☐	☒	Human research participants
☒	☐	Clinical data
☒	☐	Dual use research of concern

Methods

n/a	Involved in the study	
☒	☐	ChIP-seq
☐	☒	Flow cytometry
☒	☐	MRI-based neuroimaging

Antibodies

Antibodies used	Foci were stained with a rabbit anti-spike monoclonal antibody (BS-R2B12, GenScript A02058) at 0.5 μg/mL. Secondary goat anti-rabbit horseradish peroxidase (Abcam ab205718) antibody was added at 1 μg/mL
Validation	Information sheet for A02058 at https://www.genscript.com/antibody/A02058-MonoRab_SARS_CoV_2_Spike_S1_Antibody_BS_R2B12_mAb_Rabbit.html. Information sheet for ab205718: https://www.abcam.com/goat-rabbit-igg-hl-hrp-ab205718.html

Eukaryotic cell lines

Policy information about cell lines

Cell line source(s)	Vero E6 cells (ATCC CRL-1586) obtained from Cellonex in South Africa. The H1299-E3 cell line was derived from H1299 (CRL-5803) as described in (2) and Figure S1. H1299 cells were a gift from M. Oren, Weizmann Institute of Science.
Authentication	Cell lines have not been authenticated.
Mycoplasma contamination	The cell lines have been tested for mycoplasma contamination and are mycoplasma negative.
Commonly misidentified lines (See ICLAC register)	None.

Human research participants

Policy information about studies involving human research participants

Population characteristics	Participant characteristics are summarized in Table S1 and listed per participant in Table S2.
Recruitment	Blood samples were obtained from hospitalized adults with PCR-confirmed SARS-CoV-2 infection and/or vaccinated individuals who were enrolled in a prospective cohort study approved by the Biomedical Research Ethics Committee at the University of KwaZulu–Natal.
Ethics oversight	Study approved by the Biomedical Research Ethics Committee at the University of KwaZulu–Natal (reference BREC/00001275/2020). Use of residual swab sample was approved by the University of the Witwatersrand Human Research Ethics Committee (HREC) (ref. M210752).

Note that full information on the approval of the study protocol must also be provided in the manuscript.

Flow Cytometry

Plots

Confirm that:

☒ The axis labels state the marker and fluorochrome used (e.g. CD4-FITC).

☒ The axis scales are clearly visible. Include numbers along axes only for bottom left plot of group (a 'group' is an analysis of identical markers).

☒ All plots are contour plots with outliers or pseudocolor plots.

☒ A numerical value for number of cells or percentage (with statistics) is provided.

Methodology

Sample preparation	Plasma was separated from EDTA-anticoagulated blood by centrifugation at 500 rcf for 10 min and stored at −80°C. Aliquots of plasma samples were heat-inactivated at 56°C for 30 min and clarified by centrifugation at 10,000 rcf for 5 min.
Instrument	Plates were imaged in an ImmunoSpot Ultra-V S6-02-6140 Analyzer ELISPOT instrument with BioSpot Professional built-in image analysis (C.T.L).
Software	BioSpot Professional built-in image analysis (C.T.L).
Cell population abundance	H1299-E3 clone was previously generated and described. Abundance of infected cells with lentiviral infection was 30%/
Gating strategy	H1299-E3 clone was previously generated and described. Gating was based on FSC/SSC for live cells, then uninfected cells were used to determine mCherry positive gating.

☒ Tick this box to confirm that a figure exemplifying the gating strategy is provided in the Supplementary Information.
